# YOLO-CCG: An enhanced model for weed detection in complex cotton fields

**DOI:** 10.1371/journal.pone.0357780

**Published:** 2026-10-07

**Authors:** Lingmei Wu, Liqiang Zhang, Rifeng Wang, Huannian Meng, Yonghua Pan, Linli Jiang, Xing Zhang

**Affiliations:** 1 School of Artificial Intelligence, Guangxi Science & Technology Normal University, Laibin, Guangxi, China; 2 Guangxi Academy of Sciences, 98 Daling Road, Nanning, Guangxi, China; Hohai University, CHINA

## Abstract

Accurate weed detection is essential for site-specific herbicide application in cotton production. However, variations in weed appearance, complex field backgrounds, uneven target distributions, and leaf occlusion can result in missed detections and false positives. To address these challenges, this study proposes YOLO-CCG, an improved YOLO11-based model for weed detection in cotton fields. First, the Coordinate Attention (CA) mechanism is introduced into the backbone to embed spatial coordinate information into channel attention, thereby strengthening target localization and the representation of discriminative weed features. Second, nearest-neighbor upsampling in the neck is replaced with the Content-Aware ReAssembly of Features (CARAFE) module, which performs content-aware feature reassembly to preserve fine-grained spatial information during feature fusion. Finally, GSConv and the VoVGSCSP lightweight module are introduced into the bottom-up path of the neck to replace the corresponding standard convolution and C3k2 blocks, reducing computational complexity without increasing the parameter count. Experimental results show that, compared with YOLO11s, YOLO-CCG achieves relative improvements of 0.4%, 2.2%, 0.8%, and 1.7% in precision, recall, mAP50, and mAP50–95, respectively. These results indicate that YOLO-CCG provides an accurate and computationally efficient solution for weed detection in complex cotton-field environments.

## 1. Introduction

Cotton is one of the most important cash crops worldwide and a core raw material for the textile industry, occupying a critical position in global agricultural trade. However, weed infestation poses a substantial threat to cotton growth and yield [1]. Weeds compete with cotton seedlings for essential resources including light, water, and nutrients, provide habitats for pests and diseases, and accumulate weed seeds in the soil, all of which damage crop productivity across growing seasons [2]. Since cotton seedlings grow slowly in the early developmental stage and have weak competitiveness against weeds, ineffective or delayed weed control can cause considerable economic losses [3]. To address this practical demand, vision-based intelligent weed detection and targeted spraying technology has become a core research direction for precision cotton management, and a field robot system has been developed for seedling-stage cotton fields [4]. Accurate and efficient weed management is therefore essential for sustaining agricultural productivity and profitability, and remains a long-standing challenge in field crop production [5].

Conventional weed management strategies mainly include mechanical methods (e.g., mowing and plowing) and chemical herbicide application. Mechanical weeding is environmentally friendly and reduces herbicide dependence [6]. However, it can inflict damage on crop seedlings, particularly in densely planted fields, and often requires repeated operations due to weed regrowth [7]. Chemical control is widely adopted for its convenience and high efficiency, but overuse of herbicides has led to the emergence of resistant weed biotypes, raising growing concerns about environmental safety, food security, and human health. To address these issues, reducing herbicide usage and developing precision weeding technologies have become important trends in sustainable agriculture [8]. Precision spraying based on weed detection can effectively suppress weeds while minimizing chemical inputs, which has been proven to improve control efficiency, reduce production costs, and mitigate environmental pollution [9].

The innovative application of artificial intelligence (AI) in agriculture has catalyzed the emergence of smart agriculture as a transformative production model. As a key driver of traditional agriculture modernization, AI technology has not only reshaped core production processes but also become a critical enabler of sustainable agricultural development. Unlike experience-based traditional agriculture, smart agriculture establishes a synergistic system characterized by “precision management—intensive resource utilization—high-quality output” through deep integration of AI with agricultural production scenarios. This system significantly enhances production efficiency, lowers operational costs, and improves product quality through intelligent, full-process regulation [10]. This technological integration effectively addresses the inherent challenges of conventional agriculture—high resource consumption, low efficiency, and complex management—while providing scalable technical pathways for advancing global sustainable agriculture.

Within the precision management framework of smart agriculture, accurate weed identification and control are crucial for safeguarding crop yields. Weeds compete aggressively with crops for light, nutrients, and water, making effective management strategies indispensable. Early technical explorations in weed recognition primarily relied on computer vision combined with conventional machine learning classifiers. For example, Zhao et al. [11] utilized Hu moment invariants to extract soybean leaf features, achieving 90.5% recognition accuracy with a nearest neighbor classifier. Lavania et al. [12] combined a 3D OTSU method with principal component analysis, improving inter-row weed detection accuracy to 91.67%. Bakhshipour et al. [13] attained 92.92% classification accuracy for four common weed species in sugar beet fields using artificial neural networks. Zhang et al. [14] achieved 94.51% weed recognition accuracy in wheat fields by integrating a modified GrabCut method, an adaptive fuzzy dynamic K-means algorithm, and sparse representation classification. These early approaches demonstrated the technical feasibility of weed recognition under their respective experimental conditions, laying a foundation for subsequent research.

However, traditional computer vision methods typically identify weeds by segmenting crop images and extracting low-level features such as color, shape, and texture. Due to their multi-stage, manual design and limited generalization capacity, these methods struggle to adapt to species diversity, complex growth patterns, and variable lighting conditions encountered in field environments. As a result, they have proven inadequate for reliable weed detection under natural growing conditions.

In recent years, rapid advances in deep learning, particularly the development of object detection algorithms such as the YOLO series, have significantly alleviated the limitations of conventional agricultural perception methods [15]. Unlike the traditional “manual feature extraction + classifier” pipeline, YOLO algorithms autonomously learn discriminative features directly from large volumes of field images, exhibiting strong adaptability across varied scenarios. Deep learning models have achieved remarkable results in key areas including crop image classification, object detection, and instance segmentation [16]. These technologies enable real-time monitoring of crop growth, facilitating timely interventions. For example, the integration of computer vision with deep learning in weed identification and automated spraying systems allows precise detection and targeted chemical application. This approach has been demonstrated to reduce pesticide use and labor costs while improving weeding efficiency, offering significant benefits for both agricultural productivity and environmental protection [17,18].

In this study, we conduct a comprehensive analysis of the YOLO11 algorithm and propose a series of tailored optimization strategies to improve weed detection performance in cotton fields.

First, the Coordinate Attention (CA) mechanism [19] is incorporated into the backbone network. This module strengthens the model’s capacity to capture positional and morphological information by embedding spatial coordinate information into channel attention. Specifically, it decomposes channel attention into two separate one-dimensional feature encoding processes that aggregate features along the height and width directions, respectively. This enables the model to capture long-range dependencies along one spatial direction while preserving precise positional information along the other. The resulting feature maps are then integrated to produce direction-aware and position-sensitive attention weights, which enhance the representation of target regions.

Second, the original upsampling operator in the neck network is replaced with the Content-Aware Reassembly of Features (CARAFE) module [20]. CARAFE consists of a content-aware kernel predictor and a feature reassembly module. Its key advantage lies in its ability to adaptively generate upsampling kernels based on input feature content, enabling more effective use of contextual information. This strategy not only improves the resolution of feature maps but also enhances the model’s ability to reconstruct fine details, particularly for small weeds in complex backgrounds.

Finally, to reduce model complexity and improve feature learning efficiency, we introduce GSConv and VoVGSCSP into the bottom-up path of the neck [21]. Specifically, GSConv combines standard convolution, depthwise convolution, and channel shuffling to promote inter-channel information exchange. VoVGSCSP supports feature reuse and fusion in the corresponding C3k2 replacement blocks. Their integration reduces computational complexity without increasing the final parameter count.

The main contributions of this study are as follows:

1. We propose an improved model named YOLO-CCG for weed detection in cotton fields. The model strengthens feature representation and reduces computational complexity without increasing the number of parameters.
2. The CA mechanism is integrated into the backbone, enabling the model to jointly model channel dependencies and spatial positional information. This helps the lightweight network retain positional cues with only a small computational overhead.
3. In the neck network, nearest-neighbor upsampling is replaced with CARAFE, which predicts content-aware reassembly kernels and can preserve fine-grained local information during feature fusion.
4. The bottom-up neck path incorporates GSConv and VoVGSCSP in place of the corresponding downsampling convolution and C3k2 blocks. This reduces computational redundancy while maintaining feature fusion capacity.

## 2. Related work

### 2.1. YOLO-based weed detection methods

In recent years, the YOLO series has become one of the most widely adopted frameworks for agricultural weed detection because of its favorable balance between detection accuracy and inference speed. With the continuous evolution from YOLOv5 to YOLO12, numerous studies have introduced task-specific improvements to enhance weed detection performance under complex field conditions.

Dang et al. [22] systematically evaluated seven YOLO-based detectors on the CottonWeedDet12 dataset using Monte Carlo cross-validation. Their results showed that mAP50 ranged from 88.14% to 95.22%, while mAP50-95 ranged from 68.18% to 89.72%, demonstrating the effectiveness of YOLO-based architectures for cotton weed detection. Similarly, Khan et al. [23] evaluated YOLOv8 with multi-scale feature fusion and an anchor-free detection strategy. The model achieved 96.1% mAP50 on the CottonWeedDet12 dataset with an inference latency of 6.3 ms per frame, reduced classification errors among visually similar weed species, and used a loss function combining Complete IoU (CIoU) and Distribution Focal Loss (DFL). These studies illustrate the performance range of YOLO-based detectors for cotton weed detection.

To further improve weed detection performance, Zheng et al. [24] proposed YOLOv8-DMAS, which integrates a Dilation-wise Residual (DWR) module, a Multi-Scale module (MSBlock), and an additional small-object detection layer. Compared with YOLOv8s, the proposed model improved precision, recall, mAP50, and mAP50-95 by 1.7, 3.8, 2.1, and 3.7 percentage points, respectively. These studies demonstrate that enhancing feature extraction and multi-scale feature fusion is an effective strategy for improving weed detection performance in complex agricultural environments.

### 2.2. Attention mechanisms and feature enhancement strategies

Attention mechanisms have been extensively adopted to improve the discrimination capability of object detectors in complex agricultural scenes. By emphasizing informative regions and suppressing irrelevant background information, attention modules can significantly enhance feature representation.

Ren et al. [25] developed EY8-MFEM by incorporating Adaptive Local-Global Attention (ALGA), a Multi-scale Directional Perception Module (MDPM), and CARAFE, resulting in substantial improvements in F1-score and detection accuracy. Chen et al. [26] further proposed the YOLOv8-EGC-Fusion model, which integrates Efficient Group Convolution and Group Context Anchor Attention to balance detection accuracy and computational efficiency. Ma et al. [27] proposed YOLO-CWD by integrating an Efficient Channel-Spatial Attention (ECSA) module and a Power IoU (PIoU) loss function. Experimental results showed improvements in both detection accuracy and robustness under low-light and high-occlusion conditions.

The above studies verify that attention mechanisms and feature reconstruction strategies can effectively improve feature discrimination and model robustness under complex agricultural backgrounds, which provides a technical reference for the feature optimization design of this study.

### 2.3. Lightweight detection models for agricultural applications

Real-time agricultural applications often require deployment on resource-constrained edge devices, making lightweight model design an important research direction.

Wei et al. [28] employed C3k2_Ghost modules and a Feature Refinement Module (FRM) to construct a lightweight detector for tomato detection tasks, achieving a favorable trade-off between accuracy and efficiency in complex agricultural environments. Wang et al. [29] proposed YOLO-Weed Nano by replacing the YOLOv8n backbone with DS_HGNetV2 and introducing a LiteDetect head. The resulting model reduced the parameter count to 1.09 M (a 63.8% reduction in parameter count) while achieving 93.1% mAP50 and an inference speed of 227.3 FPS. Hu et al. [30] developed GBM-YOLOv5 for cotton impurity detection by integrating GhostBottleneck and SoftPool modules, significantly reducing computational complexity while improving detection accuracy. Likewise, Karim et al. [31] introduced the Convolutional Block Attention Module (CBAM) and C3Ghost modules into a lightweight YOLOv8 framework, improving weed recognition performance while maintaining suitability for edge deployment, and further developed a graphical user interface to support practical field operation.

For cross-crop scenarios, Li et al. [32] proposed YOLOv7-FWeed for soybean fields by introducing an improved activation function and a multi-head self-attention module. Recent studies show that lightweight convolution, feature reuse, and efficient feature fusion can reduce computational overhead while retaining competitive detection performance. These findings provide guidance for deploying weed detectors on agricultural robots and intelligent spraying platforms.

### 2.4. Research gap and motivation

Although existing studies have achieved considerable progress in agricultural weed detection, several challenges remain. First, cotton field environments are characterized by significant weed diversity, complex background interference, and frequent leaf occlusion, which continue to affect detection accuracy. Second, many high-accuracy models rely on increasingly complex network architectures, resulting in higher computational costs and reduced deployment efficiency. Third, lightweight models often sacrifice feature representation capability to achieve computational savings, leading to performance degradation when detecting small or partially occluded weeds.

Therefore, there remains a need for a detection framework that can simultaneously enhance feature representation, improve small-object perception, and maintain computational efficiency. To address these challenges, this study proposes YOLO-CCG, which integrates CA, CARAFE, GSConv, and VoVGSCSP modules to improve weed detection performance while preserving deployment feasibility in real-world agricultural applications.

## 3. Methods

### 3.1. YOLO11 model

The YOLO11 series provides five model variants: n, s, m, l, and x, with different depth and width scaling factors to adapt to different application scenarios and hardware constraints. The architecture mainly consists of three core components: backbone, neck, and head, as shown in Fig 1.

**Fig 1:**
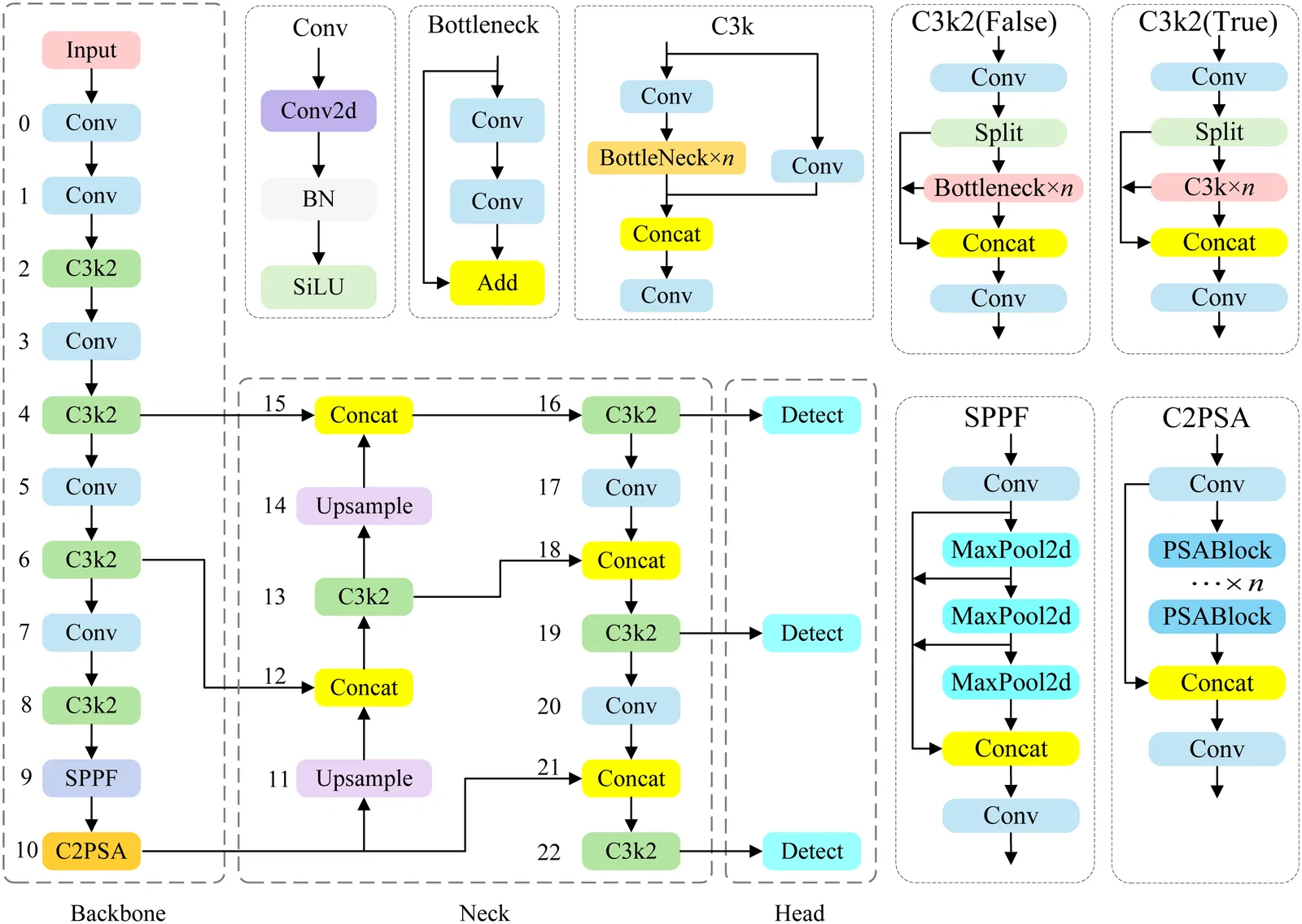
YOLO11 network structure diagram.

The backbone is responsible for multi-scale feature extraction, capturing key visual attributes such as leaf shape, color, and texture, which form the basis for subsequent target classification and localization. The neck acts as an intermediate module that receives multi-level feature maps from the backbone and performs feature fusion. It adopts a top-down and bottom-up multi-level feature fusion strategy to optimize channel information and spatial resolution, generating more robust feature representations for the detection head. This process is critical for distinguishing weeds from cotton plants under complex field conditions. Finally, the head performs weed classification and localization based on the refined feature maps. It learns category-specific features from annotated data to predict bounding boxes and category probabilities of weed targets.

Despite its strong general object detection performance, YOLO11s still faces specific challenges when applied to cotton weed detection. The high diversity of weed species and their morphological similarity to cotton seedlings (especially in color and leaf texture) increase the difficulty of accurate classification. In addition, variable field conditions such as fluctuating illumination and complex soil backgrounds further degrade detection reliability. Therefore, targeted optimizations are necessary to improve the accuracy and robustness of YOLO11s for this specific agricultural task.

### 3.2. Proposed model

To enhance the performance of the YOLO11 algorithm for cotton weed detection, this study introduces several architectural modifications based on the YOLO11s framework. First, the CA module is inserted immediately after the Spatial Pyramid Pooling Fast (SPPF) module in the backbone to enhance the model’s ability to capture position-sensitive features and improve target localization accuracy. Second, the original nearest-neighbor interpolation upsampling operators in the neck are replaced with CARAFE modules, which adaptively reconstruct fine-grained local details and contextual information during upsampling. Finally, the standard convolutional layers and C3k2 modules in the neck are substituted with GSConv and VoVGSCSP modules, respectively. These replacements streamline the feature fusion and processing pipeline, achieving a balance between improved detection performance and reduced computational complexity. The overall architecture of the proposed model, termed YOLO-CCG, is depicted in Fig 2.

**Fig 2:**
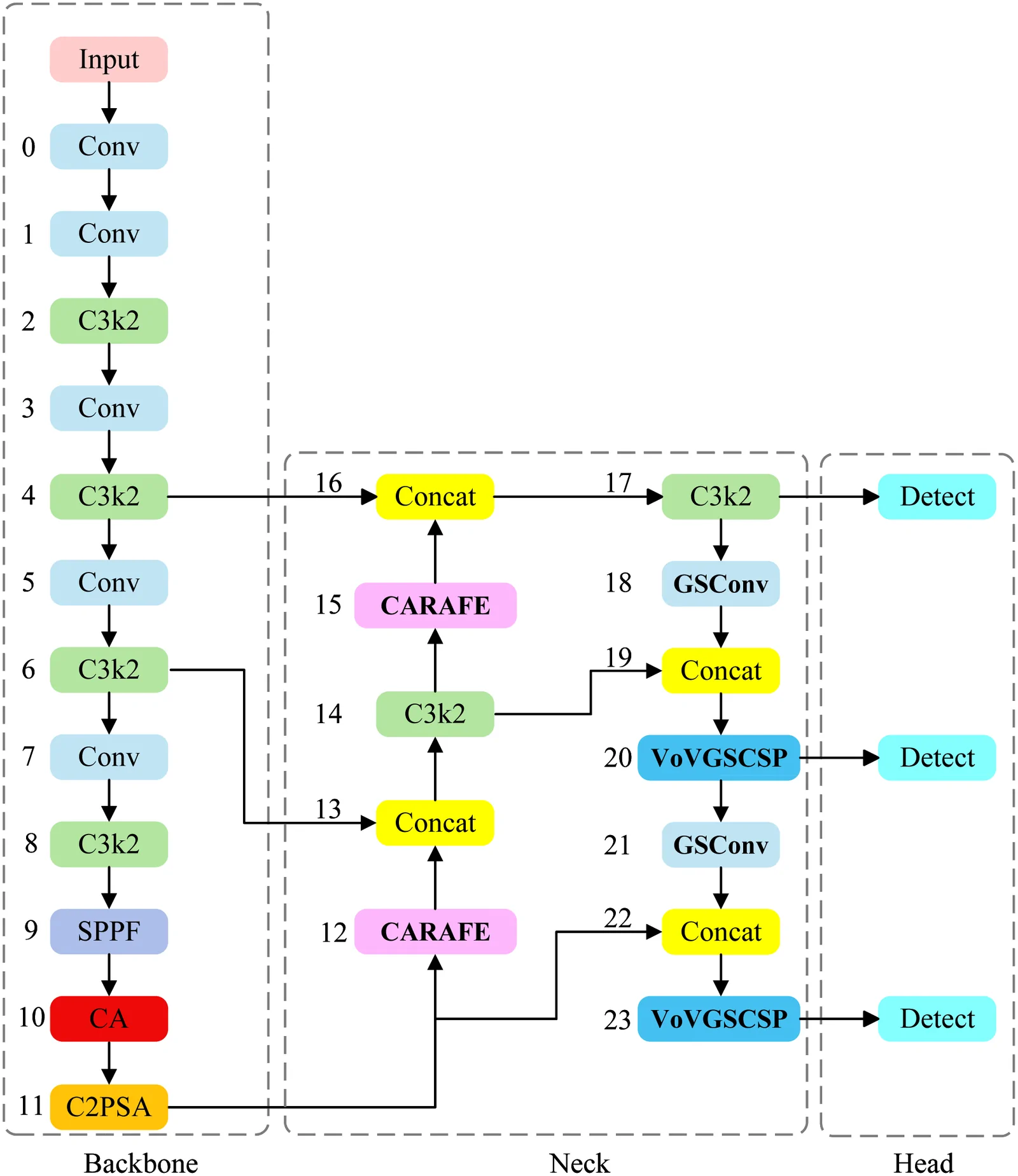
Proposed YOLO-CCG model architecture.

#### 3.2.1. Coordinate attention.

In natural field environments, crop cultivation and weed growth often involve challenges such as overlapping foliage and spatial occlusion. These factors impose stringent demands on detection models, particularly regarding localization precision and the capability to accurately capture target shapes. Conventional approaches often enhance feature representation by increasing model parameters, which leads to a significant increase in computational complexity and can dilute the model’s focus on salient features. Attention mechanisms can improve model performance by suppressing background noise and highlighting key regions without substantially increasing computational overhead. Nonetheless, conventional attention modules, such as Squeeze-and-Excitation (SE) and Convolutional Block Attention Module (CBAM), may compromise precise positional information when encoding channel weights and struggle to effectively capture long-range dependencies. To address these issues, we incorporate the CA mechanism into the YOLO11 framework. CA factorizes two-dimensional global pooling into two parallel one-dimensional feature encoding operations along the height and width dimensions. Together, these operations capture long-range dependencies along one spatial direction while retaining precise positional information along the other. The resulting attention maps are direction-aware and position-sensitive, helping the network delineate weed contours and localize weed instances in complex field scenes. The structure of CA is illustrated in Fig 3.

**Fig 3:**
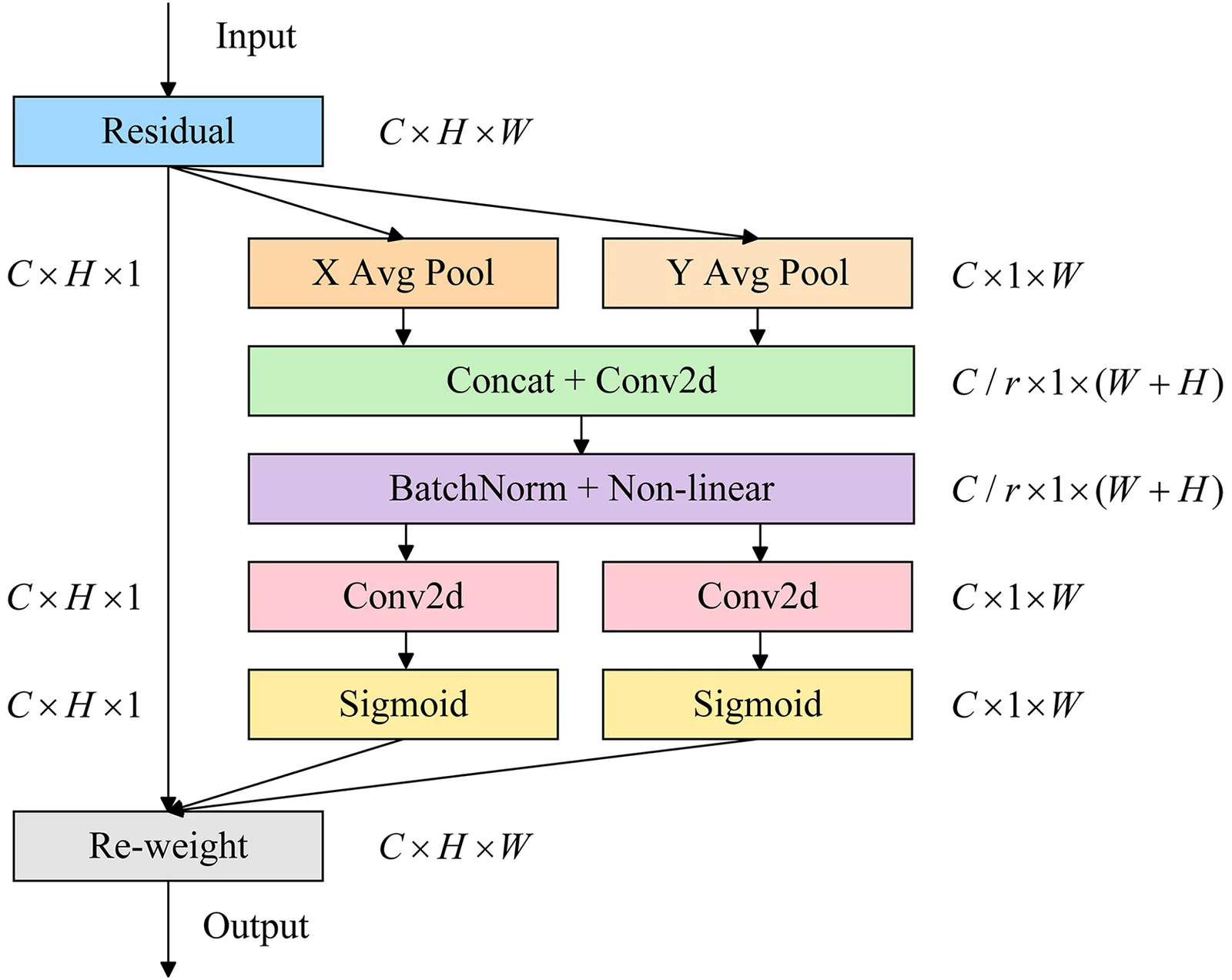
Schematic of Coordinate Attention.

Given an input feature tensor $X\in R^{C\times H\times W}$, where C,H, and W denote the number of channels, height, and width of the feature map, respectively, the Coordinate Attention mechanism first performs one-dimensional global average pooling along the horizontal and vertical dimensions to extract spatial coordinate information. Specifically, pooling along the width dimension for each height coordinate generates the height-oriented feature map $z^{h}\in R^{C\times H}$, as shown in Eq (1):


$$\begin{array}{c} z_{c}^{h}\left(h\right)=\frac{\text{1}}{W}\sum_{0\leq i<W}x_{c}\left(h,i\right) \end{array}$$ (1)

where $z_{c}^{h}\left(h\right)$ represents the pooled output for the c-th channel at height h, and $x_{c}\left(h,i\right)$ denotes the feature value at spatial location (h,i).

Similarly, pooling along the height dimension for each width coordinate generates the width-oriented feature map $z^{w}\in R^{C\times W}$, as shown in the following Eq (2):


$$\begin{array}{c} z_{c}^{w}\left(w\right)=\frac{\text{1}}{H}\sum_{0\leq j<H}x_{c}\left(j,w\right) \end{array}$$ (2)

where $z_{c}^{w}\left(w\right)$ represents the pooled output for the *c*-th channel at width w.

Subsequently, the two feature maps $z^{h}$ and $z^{w}$ are concatenated along the spatial dimension and passed through a shared bottleneck transformation. This process can be formulated as shown in Eq (3):


$$\begin{array}{c} f=\delta \left(F_{\text{1}}\left(\left[z^{h},z^{w}\right]\right)\right) \end{array}$$ (3)

where $\left[z^{h},z^{w}\right]$ denotes the concatenation, $F_{\text{1}}$ is a 1 × 1 convolutional transformation function, and δ is a non-linear activation function. The bottleneck channel dimension is C/r. Here, r is the reduction ratio. The resulting intermediate feature map $f\in R^{C/r\times (H+W)}$ is then split along the spatial dimension into two tensors, $f^{h}\in R^{C/r\times H}$ and $f^{w}\in R^{C/r\times W}$.

Two additional 1 × 1 convolutional transformations $F_{h}$ and $F_{w}$ are applied to $f^{h}$ and $f^{w}$, respectively, to restore the channel number to C. The corresponding height and width attention weights are then generated using the sigmoid activation function, as shown in Eqs (4) and (5):


$$\begin{array}{c} g^{h}=\sigma \left(F_{h}\left(f^{h}\right)\right) \end{array}$$ (4)

$$\begin{array}{c} g^{w}=\sigma \left(F_{w}\left(f^{w}\right)\right) \end{array}$$ (5)

where σ is the sigmoid function, $g^{h}\in R^{C\times H}$, $g^{w}\in R^{C\times W}$.

The final output Y is obtained by element-wise multiplying the input tensor X by the attention weights $g^{h}$ and $g^{w}$, as shown in Eq (6):


$$\begin{array}{c} y_{c}\left(i,j\right)=x_{c}\left(i,j\right)\times g_{c}^{h}\left(i\right)\times g_{c}^{w}\left(j\right) \end{array}$$ (6)

where $y_{c}\left(i,j\right)$ is the value of the output feature map at spatial location (i,j).

By explicitly embedding coordinate information into channel attention, the CA module enhances the model’s ability to capture long-range dependencies while maintaining a lightweight structure. This significantly improves the perception of target positions and shapes, providing an effective solution for the accurate detection of weeds in natural scenes.

#### 3.2.2. CARAFE.

The original neck module of YOLO11 utilizes nearest-neighbor interpolation for upsampling. This method, which determines the upsampling kernel based solely on pixel spatial positions without considering the semantic content of the feature maps, suffers from a limited receptive field and fails to capture rich contextual information. To address this limitation, we replaced the standard upsampling method with the CARAFE operator. As illustrated in Fig 4, the CARAFE algorithm consists of two core components: a kernel prediction module that generates adaptive upsampling kernels based on local content, and a content-aware reassembly module that performs the feature reassembly using these predicted kernels.

**Fig 4:**
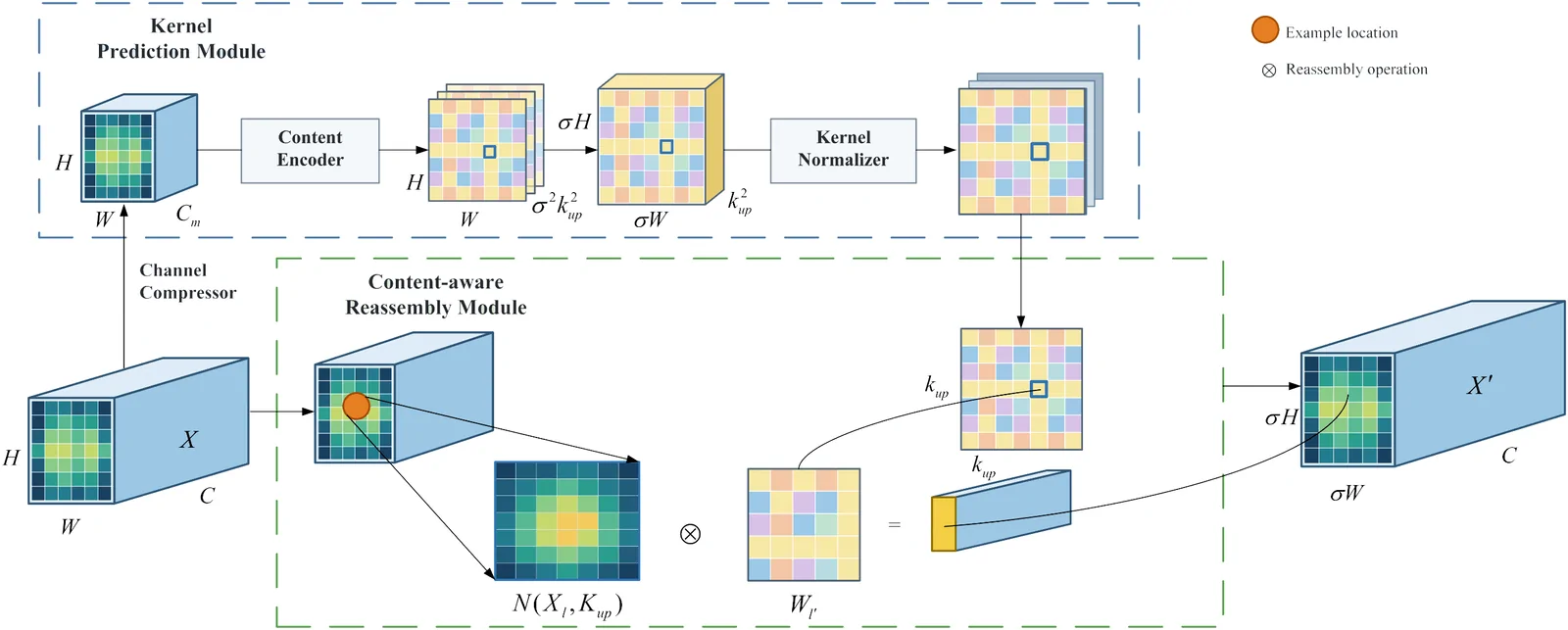
Schematic of CARAFE.

Given an input feature map X of shape H×W×C and an upsampling ratio σ, where H,W and C denote height, width, and the number of channels, respectively, CARAFE first employs the kernel prediction module to generate the upsampling kernels and then utilizes the content-aware reassembly module to produce the output feature map $X^{\prime }$ of size σH×σW×C.

In the kernel prediction module, the input feature map is first processed by a 1 × 1 convolution to compress the channel dimension from C to $C_{m}$. Subsequently, a convolutional layer with a kernel size of $k_{encoder}\times k_{encoder}$ and a stride of 1 is applied, yielding a feature map with $\sigma^{\text{2}}k_{up}^{\text{2}}$ channels, where $k_{up}$ is the reassembly kernel size. This output is then reorganized to form the adaptive upsampling kernels with a shape of $\sigma H\times \sigma W\times k_{up}^{\text{2}}$. These dynamically generated kernels are content-aware, enabling the capture of more precise contextual information. Finally, the kernels are normalized via a softmax function to ensure that the sum of the weights equals one.

The content-aware reassembly module is responsible for the actual upsampling operation. For each target location $l^{\prime }$, the module extracts a $k_{up}\times k_{up}$ neighborhood $N\left(X_{l},k_{up}\right)$ centered at the corresponding source location l in the input feature map. The reassembly is achieved by performing a matrix multiplication between the unfolded neighborhood $N\left(X_{l},k_{up}\right)$ and the normalized kernel $W_{l^{\prime }}$, ultimately generating the new, upsampled feature map $X^{\prime }$ of size σH×σW×C.

Through its content-aware mechanism, CARAFE adaptively learns the optimal sampling strategy for local regions, thereby effectively extracting detailed texture and contextual semantic information. Compared to conventional upsampling methods, CARAFE benefits from a larger and more adaptive receptive field, producing feature maps that are richer in information and thus providing superior feature representations for subsequent detection tasks.

#### 3.2.3. GSConv and VoVGSCSP.

GSConv integrates the advantages of both standard convolution and depthwise convolution (DWConv) [21,33]. As illustrated in Fig 5, the standard convolutional layer is first applied to the input feature map to produce $C_{\text{2}}/2$ channels. A depthwise convolution is subsequently applied to this intermediate feature map to generate another $C_{\text{2}}/2$ channels. The two feature maps are concatenated and channel-shuffled [34], producing an output with $C_{\text{2}}$ channels. Thus, GSConv combines the feature representation capability of standard convolution with the low computational cost of depthwise convolution.

**Fig 5:**
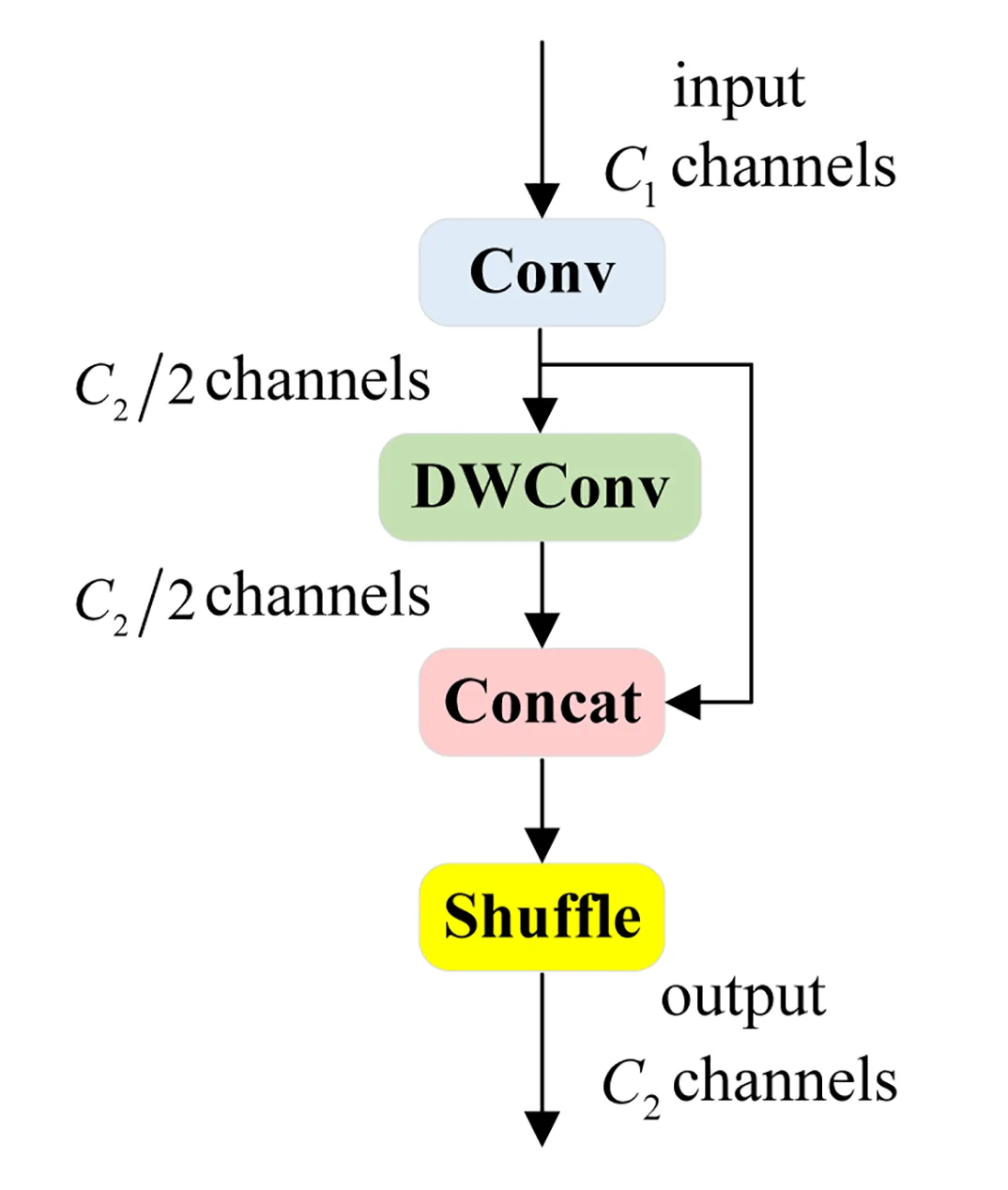
Structure of GSConv.

In the neck network, we adopt a Slim-Neck design by replacing the original convolutional modules and C3k2 modules with GSConv and VoVGSCSP modules, respectively, to improve feature fusion efficiency and detection accuracy. The structure of the GS bottleneck is shown in Fig 6(a). The input is processed by two branches: one passes through two sequential GSConv modules, and the other is processed by a single standard convolution module. The outputs of these two branches are then added by element-wise summation to form the final output.

**Fig 6:**
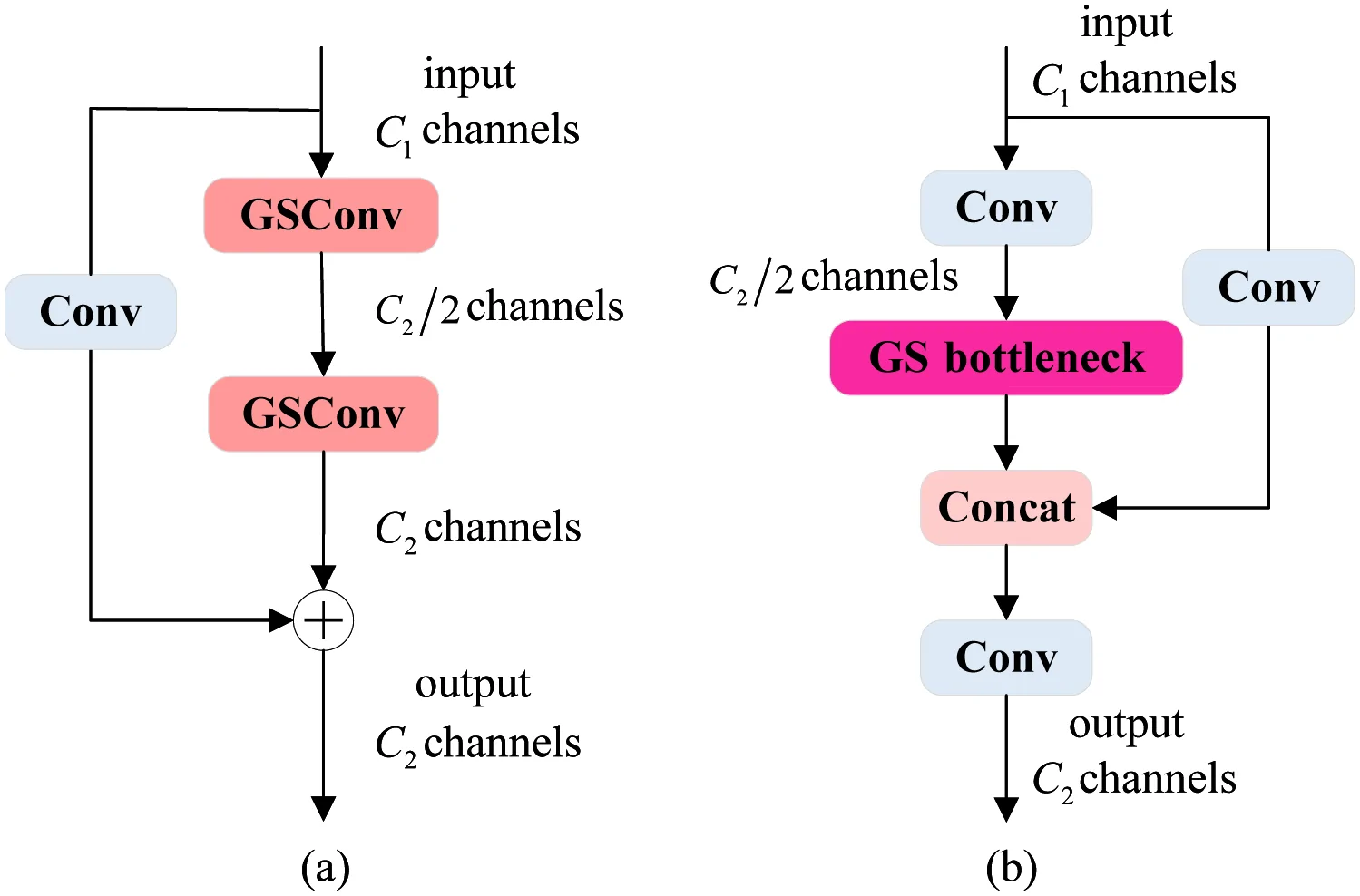
GS bottleneck and VoVGSCSP modules.

Building upon the GS bottleneck, the VoVGSCSP module is introduced to replace the original C3k2 module in YOLO11, thereby enhancing feature extraction capacity. Its architecture is depicted in Fig 6(b). The input is first processed by two parallel convolutional layers, each producing half the number of target output channels. One branch serves as a shortcut connection directly to the concatenation layer, while the other undergoes feature extraction through a GSBottleneck module. The features from both branches are then concatenated along the channel dimension and fused by a final convolutional layer.

The integration of GSConv and VoVGSCSP modules not only reduces the model’s computational burden but also enhances its feature representation capacity and multi-scale perceptual ability for target regions.

## 4. Experiments and analysis

To evaluate the performance of the proposed model, we first conducted ablation studies to quantify the contribution of each component and their combined effect. We then compared the proposed model with representative detectors using the same evaluation metrics. Finally, qualitative visualizations were examined to complement the quantitative results.

### 4.1. Dataset

This study employed the public CottonWeedDet12 dataset [22] for all experiments. The dataset contains 5,648 RGB images with 9,370 annotated bounding boxes in total. These images were captured using smartphones or handheld digital cameras under natural lighting conditions between June and September 2021, covering diverse illumination scenarios, weed growth stages, weather conditions, and field locations. The dataset includes twelve distinct weed categories, with all images stored in JPEG format. For consistency, all images were uniformly resized to 640 × 640 pixels with aspect-ratio-preserving padding. To robustly evaluate the model’s performance and generalization capability, the dataset was randomly split into training, validation, and test sets with ratios of 75%, 15%, and 10%, respectively. The specific weed categories are presented in Fig 7.

**Fig 7:**
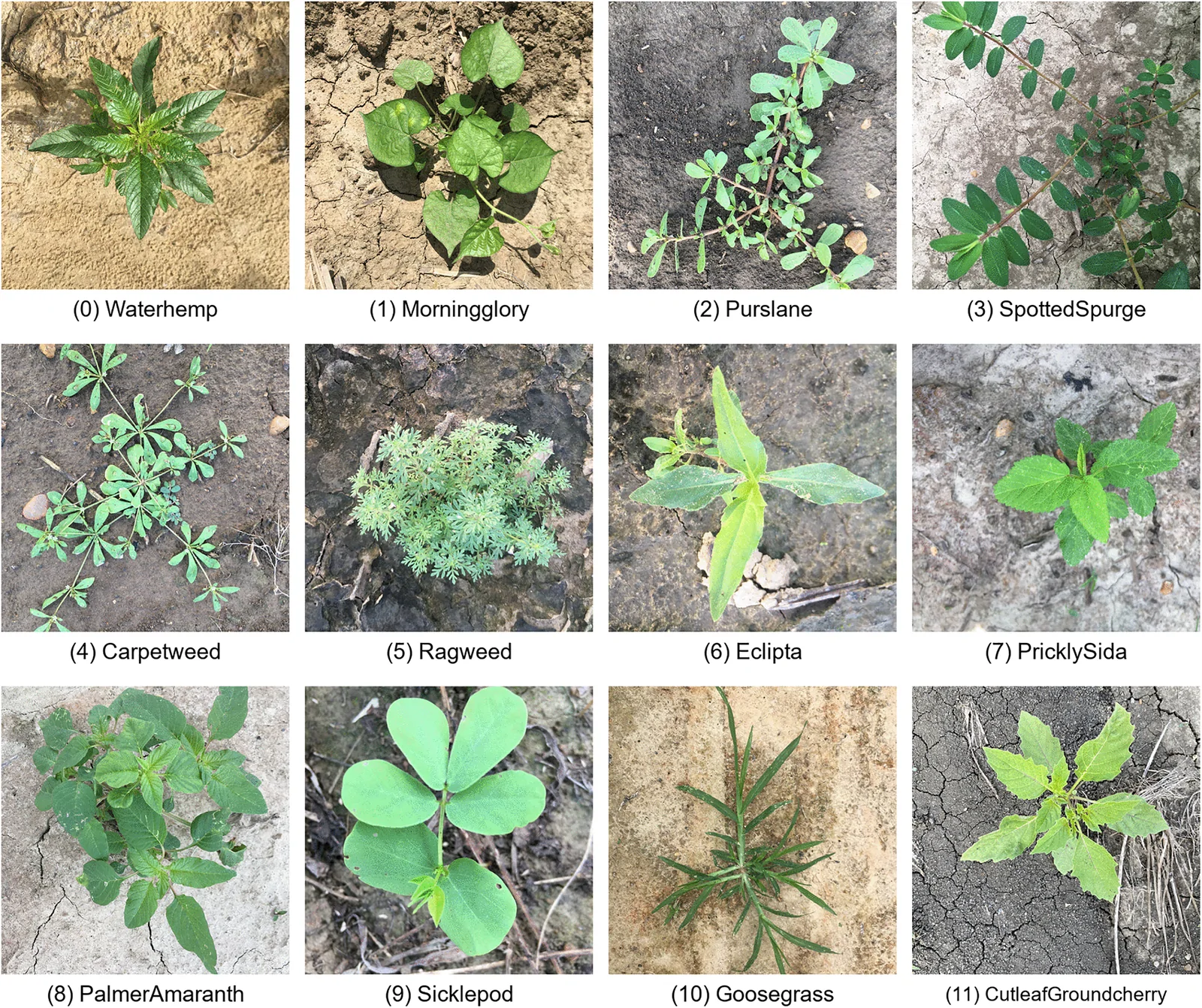
Example images from the CottonWeedDet12 dataset.

### 4.2. Experimental configuration

All experiments were conducted on a workstation running Ubuntu 22.04 OS. Model training and inference were performed on an NVIDIA GeForce RTX 4090D GPU with 24 GB VRAM, equipped with 80 GB system RAM. The deep learning framework was PyTorch 2.5.1, and all code was implemented in Python 3.12 with CUDA 12.4. The detailed hardware and software environment is summarized in Table 1, and the training hyperparameters are listed in Table 2.

**Table 1:** Experimental platform settings.

Configuration	Value
OS	Ubuntu 22.04
GPU	RTX 4090D (24 GB)
CUDA Version	12.4
Python	3.12
Deep learning framework	PyTorch 2.5.1

**Table 2:** Training hyperparameters.

Parameter	Value
Learning rate	0.01
Epochs	200
Batch size	16
Optimizer	SGD
Early stopping patience	50
Number of workers	8
Momentum	0.937
Mosaic disabled in final epochs (close_mosaic)	10

### 4.3. Evaluation metrics

To comprehensively evaluate the performance and efficiency of the model in object detection tasks, this study adopts precision (P), recall (R), mean average precision (mAP), the number of model parameters (Params), computational complexity in giga floating-point operations (GFLOPs), model size and frames per second (FPS) as core evaluation metrics.

Precision measures the proportion of true positive samples among all positive predictions, reflecting the model’s ability to suppress false positives. A higher precision indicates fewer false-positive detections. It is calculated as shown in Eq (7). Recall measures the proportion of correctly detected positive samples among all real positive samples, reflecting the ability to avoid missed detections, as defined in Eq (8).


$$\begin{array}{c} Precision=\frac{TP}{TP+FP} \end{array}$$ (7)

$$\begin{array}{c} Recall=\frac{TP}{TP+FN} \end{array}$$ (8)

Here, *TP* (true positives) is the number of predicted weed boxes correctly matched to ground-truth objects, *FP* (false positives) is the number of predicted boxes not matched to a ground-truth object under the specified matching criterion, and *FN* (false negatives) is the number of ground-truth objects that are not detected.

Mean average precision (mAP) is the key metric for object detection performance. First, average precision (AP) for each category is calculated by integrating the precision-recall (P-R) curve over all recall levels, as expressed in Eq (9). mAP is then obtained by averaging AP values across all categories, as shown in Eq (10), where N is the number of object categories. In this study, mAP50 and mAP50-95 are used as the main evaluation criteria: mAP50 refers to the mAP at an Intersection over Union (IoU) threshold of 0.5, whereas mAP50-95 represents the average mAP over IoU thresholds from 0.5 to 0.95 with a step size of 0.05. Higher mAP indicates better overall detection accuracy.


$$\begin{array}{c} AP=\int_{\text{0}}^{\text{1}}P\left(R\right)dR \end{array}$$ (9)

$$\begin{array}{c} mAP=\frac{\text{1}}{N}\sum_{i=1}^{N}AP_{i} \end{array}$$ (10)

Detection speed is quantified in frames per second (FPS), which represents the average number of input images processed by the model per second. A higher FPS indicates lower inference latency and superior real-time detection capability.

### 4.4. Ablation experiments

To validate the individual contributions of the proposed modules and their combined effects, ablation studies were conducted on the YOLO11s baseline. The CA, CARAFE, and GSConv + VoVGSCSP modules were incrementally integrated, and their performance was assessed using P, R, mAP50, mAP50-95, Params, GFLOPs, model size, and FPS. In Table 3, “√” indicates that the corresponding module was included; blank cells indicate that it was not included.

**Table 3:** Results of the ablation experiments on the test set.

YOLO11s	CA	CARAFE	GSConv + VoVGSCSP	P (%)	R (%)	mAP50 (%)	mAP50-95 (%)	Params (M)	GFLOPs	Size (MB)	FPS
✓				94.4	88.1	94.3	88.5	9.4	21.3	19.2	212.8
✓	✓			96.0	88.2	94.5	89.1	9.4	21.4	19.2	243.9
✓		✓		95.4	89.0	94.8	88.8	9.6	21.6	19.5	200.0
✓			✓	95.2	88.3	94.5	88.9	9.3	20.2	18.9	285.7
✓	✓	✓		93.7	89.8	95.3	89.4	9.5	21.5	19.4	232.6
✓	✓	✓	✓	94.8	90.0	95.1	90.0	9.4	20.4	19.1	204.1

As shown in Table 3, the baseline YOLO11s model achieved a precision of 94.4%, recall of 88.1%, mAP50 of 94.3%, and mAP50-95 of 88.5%, with 9.4 M parameters, 21.3 GFLOPs, a model size of 19.2 MB, and an inference speed of 212.8 FPS.

The first set of experiments introduced the CA module into YOLO11s. Compared with the baseline model, the CA variant improved precision, recall, mAP50, and mAP50-95 by 1.7%, 0.1%, 0.2% and 0.7%, respectively. Meanwhile, the parameter count remained unchanged at 9.4 M, while GFLOPs increased slightly by 0.1 GFLOPs. These results indicate that the CA module can effectively enhance feature representation with negligible computational overhead.

The second set of experiments replaced the original upsampling operation with the CARAFE module. Compared with the baseline model, the CARAFE variant improved precision, recall, mAP50, and mAP50-95 by 1.1%, 1.0%, 0.5% and 0.3%, respectively. However, the parameter count increased to 9.6 M and GFLOPs increased to 21.6 GFLOPs, resulting in a slight reduction in inference speed. This shows that CARAFE can improve feature reconstruction capability and enhance detection performance.

The third set of experiments replaced the standard convolution and C3k2 modules in the neck network with GSConv and VoVGSCSP, respectively. Compared with the baseline model, the GSConv + VoVGSCSP variant improved precision, recall, mAP50, and mAP50-95 by 0.8%, 0.2%, 0.2%, and 0.5%, respectively. Meanwhile, the parameter count decreased from 9.4 M to 9.3 M and GFLOPs decreased from 21.3 GFLOPs to 20.2 GFLOPs. In addition, the inference speed increased significantly to 285.7 frames/s. These results indicate that GSConv and VoVGSCSP effectively reduce computational complexity while maintaining competitive detection accuracy.

The fourth set of experiments combined the CA and CARAFE modules. Compared with the baseline model, the CA+CARAFE variant decreased precision by 0.7% and improved recall, mAP50, and mAP50-95 by 1.9%, 1.1%, and 1.0%, respectively. This result indicates that the combination of attention enhancement and content-aware upsampling can strengthen feature extraction and localization capability, although a slight trade-off in precision was observed.

The final experiment corresponds to the proposed YOLO-CCG model, which integrates the CA, CARAFE, GSConv, and VoVGSCSP modules. Compared with the baseline model, YOLO-CCG improved precision, recall, mAP50, and mAP50-95 by 0.4%, 2.2%, 0.8%, and 1.7%, respectively. At the same time, the parameter count remained unchanged at 9.4 M, while GFLOPs decreased by 4.2%. Although the inference speed was 4.1% lower than that of the baseline model and lower than that of the GSConv-based lightweight model, YOLO-CCG achieved consistent improvements across all four accuracy metrics while maintaining a lightweight architecture. These results demonstrate that the complementary integration of CA, CARAFE, GSConv, and VoVGSCSP improves feature representation, feature fusion, and multi-scale detection capability, thereby enhancing overall detection performance. Unless otherwise specified, the percentage changes reported in this section are calculated relative to the baseline YOLO11s model.

### 4.5. Comparison with other models

The proposed YOLO-CCG was further compared with several representative state-of-the-art (SOTA) detectors to assess its effectiveness for cotton weed detection. The evaluation metrics are consistent with the ablation study. Detailed results are shown in Table 4.

**Table 4:** Comparison of different models.

Model	P (%)	R (%)	mAP50 (%)	mAP50- 95 (%)	Params (M)	GFLOPs	Size (MB)	FPS
RT-DETR-ResNet50 [35]	94.2	88.3	93.5	87.2	41.9	125.7	86.1	133.3
YOLOv5s [36]	91.7	88.4	94.6	86.9	9.12	23.9	18.5	238.1
YOLOv6s [37]	91.8	91.0	94.8	88.3	16.3	44.0	32.8	263.2
YOLOv8s [38]	93.8	88.9	94.8	88.5	11.0	28.5	22.5	357.1
YOLOv9s [39]	93.6	88.4	95.0	89.8	7.2	26.8	15.2	250.0
YOLOv10s [40]	93.5	89.4	94.7	88.1	7.2	24.5	16.5	434.8
YOLO11n [41]	92.0	88.0	93.6	86.4	2.6	6.3	5.5	270.3
YOLO11s [41]	94.4	88.1	94.3	88.5	9.4	21.3	19.2	212.8
YOLO12n [42]	92.6	88.3	94.1	86.6	2.6	6.3	5.5	181.8
YOLO12s [42]	93.3	89.1	94.8	89.2	9.2	21.2	18.9	188.7
YOLO-CCG (ours)	94.8	90.0	95.1	90.0	9.4	20.4	19.1	204.1

The comparative results presented in Table 4 indicate that the proposed YOLO-CCG model achieves an excellent balance between detection accuracy and computational efficiency when compared with representative state-of-the-art object detection models.

Specifically, YOLO-CCG achieves a precision of 94.8% and a recall of 90.0%, outperforming most lightweight detectors such as YOLO11n (92.0%, 88.0%) and YOLO11s (94.4%, 88.1%) while maintaining competitive precision. In terms of mAP50, YOLO-CCG reaches 95.1%, exceeding YOLO11s (94.3%) and YOLOv8s (94.8%), and slightly surpassing YOLOv9s (95.0%). For mAP50-95, YOLO-CCG achieves 90.0%, which is higher than YOLOv9s (89.8%) and YOLO12s (89.2%), indicating superior localization performance across different IoU thresholds.

The superior performance of YOLO-CCG can be attributed to the complementary effects of the proposed modules in addressing several key challenges of cotton weed detection. In practical field environments, weed targets often exhibit significant variations in size, complex background interference, and partial occlusion caused by cotton leaves. These factors increase the difficulty of accurate target localization and classification.

The Coordinate Attention module enhances feature representation by simultaneously modeling channel relationships and long-range positional information. This mechanism enables the network to focus more effectively on discriminative weed regions while suppressing irrelevant background information. As a result, the model is better able to distinguish weeds from visually similar crop leaves and surrounding vegetation.

The CARAFE upsampling operator further improves feature reconstruction during multi-scale feature fusion. Unlike conventional interpolation-based upsampling methods, CARAFE generates content-aware kernels according to local semantic information, allowing fine-grained features to be preserved during feature propagation. This capability is particularly beneficial for detecting small weed targets and partially occluded weeds that frequently appear in cotton fields.

In addition, GSConv and VoVGSCSP contribute to a lightweight design by reducing redundant computation while maintaining effective feature extraction and fusion. This combination improves computational efficiency without substantially limiting feature diversity.

In terms of computational complexity, YOLO-CCG contains 9.4 M parameters and requires 20.4 GFLOPs. Its computational complexity is 4.2% lower than that of YOLO11s and substantially lower than those of YOLOv6s and RT-DETR-ResNet50. Its inference speed of 204.1 FPS remains compatible with real-time processing on the evaluated platform.

From an application perspective, agricultural intelligent spraying systems and field robots often operate on resource-constrained edge devices that require both real-time processing capability and high detection accuracy. The lightweight design of YOLO-CCG suggests potential for meeting these deployment requirements while maintaining robust weed detection performance under complex field conditions.

Overall, YOLO-CCG achieves a favorable trade-off among detection accuracy, computational efficiency, and deployment feasibility, demonstrating strong potential for real-time cotton weed detection and precision agriculture applications.

### 4.6. Comparison of different attention mechanisms

To quantitatively validate the advantages of the CA module for cotton weed detection, this study compared five common attention mechanisms: Squeeze-and-Excitation (SE), Efficient Multi-scale Attention (EMA), Efficient Channel Attention (ECA), Convolutional Block Attention Module (CBAM), and Global Attention Mechanism (GAM) [43–47]. SE compresses channel features through global average pooling and then learns channel weights via a fully connected layer to enhance critical channels. EMA splits features into groups and applies parallel 1 × 1 and 3 × 3 convolutions to achieve cross-dimension interaction and multi-scale aggregation at minimal computational cost. ECA employs one-dimensional convolutions to capture inter-channel dependencies, which reduces parameters and improves efficiency. CBAM is an attention mechanism that integrates spatial and channel attention. GAM significantly enhances the performance of deep neural networks in cross-dimensional interactions by preserving and strengthening both channel and spatial information. All models were trained under identical settings. The impact of different attention mechanisms on model performance is shown in Table 5.

**Table 5:** Performance comparison of different attention mechanisms based on YOLO11s.

Model	P (%)	R (%)	mAP50 (%)	mAP50- 95 (%)	Params (M)	GFLOPs	Size (MB)	FPS
YOLO11s	94.4	88.1	94.3	88.5	9.4	21.3	19.2	212.8
YOLO11s-SE	92.8	88.5	94.6	88.9	9.5	21.4	19.3	232.6
YOLO11s-EMA	93.6	86.7	95.2	89.2	9.6	21.6	19.3	200.0
YOLO11s-ECA	94.5	88.0	95.1	88.7	9.4	21.3	19.2	243.9
YOLO11s-CBAM	96.1	88.0	95.2	89.8	9.7	21.5	19.7	232.6
YOLO11s-GAM	94.8	88.1	94.7	88.5	16.0	26.6	32.3	250.0
YOLO11s-CA (ours)	96.0	88.2	94.5	89.1	9.4	21.4	19.2	243.9

As shown in Table 5, different attention mechanisms exhibit distinct advantages in cotton weed detection. Compared with the baseline YOLO11s model, all attention mechanisms improve at least one evaluation metric, indicating that attention mechanisms effectively enhance feature representation capability.

Among the compared methods, CBAM achieved the highest precision (96.1%) and mAP50-95 (89.8%) and tied with EMA for the highest mAP50 (95.2%). This gain was accompanied by increases in parameter count, model size, and computational complexity relative to the baseline.

Compared with the baseline YOLO11s model, the CA variant achieved 96.0% precision, 88.2% recall, 94.5% mAP50, and 89.1% mAP50-95 with 9.4 M parameters and 21.4 GFLOPs. Although CA did not maximize every metric, it improved precision and mAP50-95 with negligible additional computational cost and was therefore selected for YOLO-CCG.

In particular, Coordinate Attention preserves long-range positional information while simultaneously modeling channel dependencies, allowing the network to focus more effectively on weed regions under complex field environments. This characteristic is especially beneficial for distinguishing target weeds from visually similar background vegetation and crop leaves.

Therefore, considering both detection performance and lightweight deployment requirements, the CA module was selected as the attention mechanism for the proposed YOLO-CCG model.

### 4.7. Statistical significance analysis

Tables 3–5 report results from individual runs, whereas Table 6 summarizes five paired runs. A statistical analysis was performed to evaluate whether the observed performance improvements were statistically significant rather than attributable to random fluctuations. Five paired training runs were conducted for YOLO11s and YOLO-CCG using the same random seeds for both the baseline and the proposed model. The mean and standard deviation of each metric are reported, and the one-sided Wilcoxon signed-rank test was applied for significance analysis at alpha = 0.05, with the alternative hypothesis that YOLO-CCG outperformed YOLO11s. Results are shown in Table 6.

**Table 6:** Statistical comparison between YOLO11s and YOLO-CCG.

Model	P (%)	R (%)	mAP50 (%)	mAP50- 95 (%)	Params (M)	GFLOPs	Size (MB)	FPS
YOLO11s (Baseline)	92.78 ± 1.11	89.14 ± 0.78	94.44 ± 0.35	88.38 ± 0.43	9.4 ± 0.0	21.3 ± 0.0	19.2 ± 0.0	232.08 ± 74.94
YOLO-CCG (ours)	94.76 ± 1.06*	89.20 ± 0.58	95.00 ± 0.37*	89.24 ± 0.48*	9.4 ± 0.0	20.4 ± 0.0	19.1 ± 0.0	197.86 ± 35.46
P-value (Wilcoxon)	0.0313	0.6875	0.0313	0.0313	–	–	–	0.8438
Significance	p < 0.05	ns	p < 0.05	p < 0.05	–	–	–	ns

Note: * indicates a statistically significant difference at p < 0.05; ns indicates no statistically significant difference (*p* ≥ 0.05).

Statistical analysis using the Wilcoxon signed-rank test suggests that the proposed YOLO-CCG model achieves statistically significant improvements in precision (*p* = 0.0313), mAP50 (*p* = 0.0313), and mAP50-95 (*p* = 0.0313), all satisfying the significance criterion of *p* < 0.05. In contrast, no statistically significant difference is observed for recall (*p* = 0.6875) or FPS (*p* = 0.8438).

Furthermore, the standard deviations of most performance metrics are reduced in the proposed model, suggesting improved robustness and prediction stability. These findings suggest that the performance gains achieved by YOLO-CCG are not incidental but result from the integration of the CA, CARAFE, GSConv, and VoVGSCSP modules.

Overall, the proposed model achieves statistically validated improvements in detection accuracy while maintaining a lightweight architecture and acceptable inference efficiency.

### 4.8. Visualization analysis

#### 4.8.1. Confusion matrix.

To graphically illustrate the performance of YOLO-CCG for each weed category, we plotted a column-normalized confusion matrix on the test set, as shown in Fig 8. The horizontal axis represents true labels, and the vertical axis represents predicted labels. Diagonal entries represent correct classifications, off-diagonal entries indicate inter-class confusion, the background row represents false negatives, and the background column represents false positives. The matrix shows high consistency between predicted and true labels for most categories, indicating that the model achieves reliable classification performance for weed recognition.

**Fig 8:**
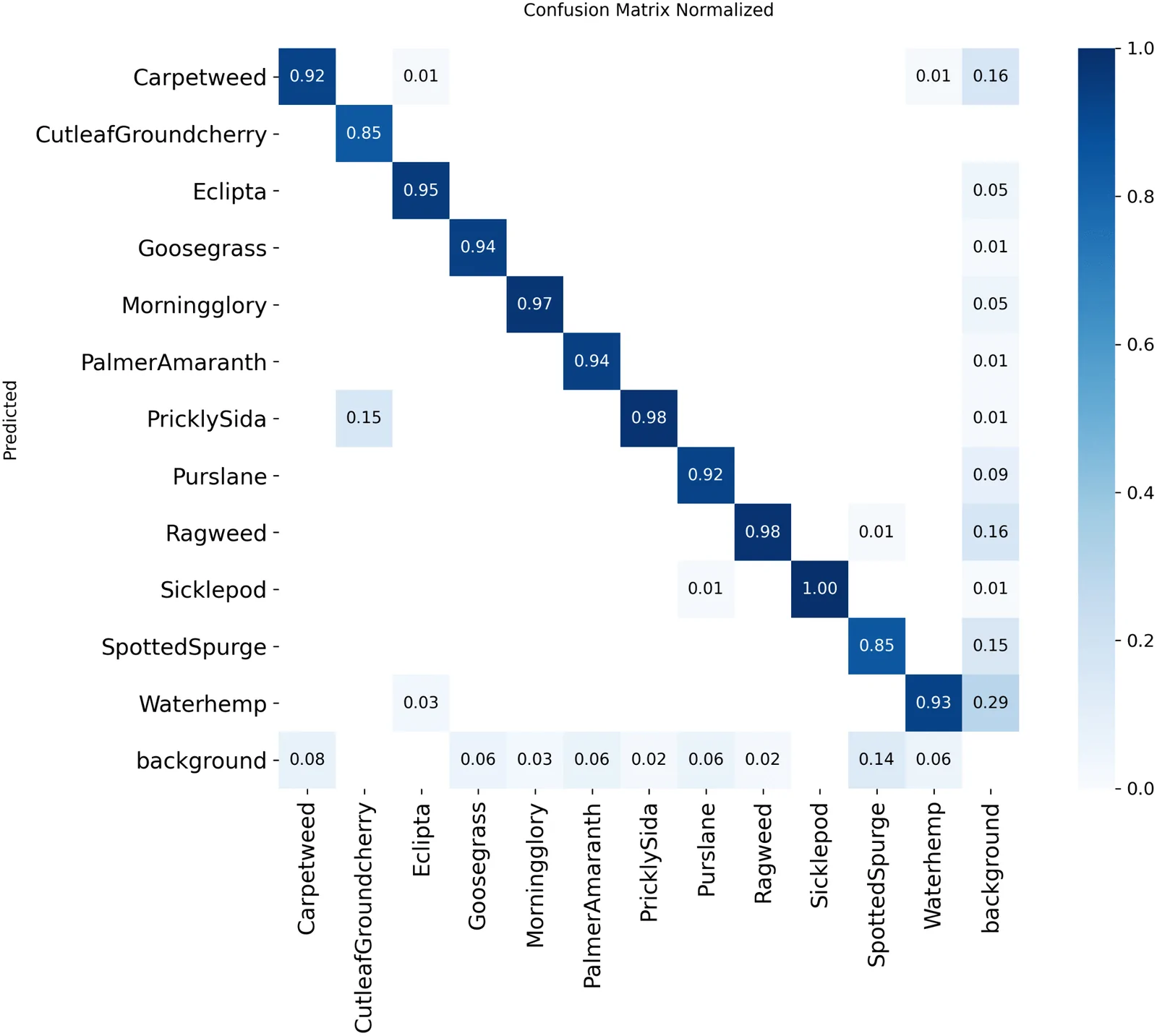
Confusion matrix of YOLO-CCG on the test set.

#### 4.8.2. Detection result visualization.

Detection-result visualization provides a qualitative comparison of target localization, object size, and predicted category. Representative test images containing complex backgrounds, dense weeds, multiple species, and small targets are shown in Fig 9. In these examples, YOLO-CCG exhibits fewer missed detections of small or occluded weeds and fewer false positives caused by background clutter than YOLO11s, demonstrating more robust feature discrimination in challenging scenarios.

**Fig 9:**
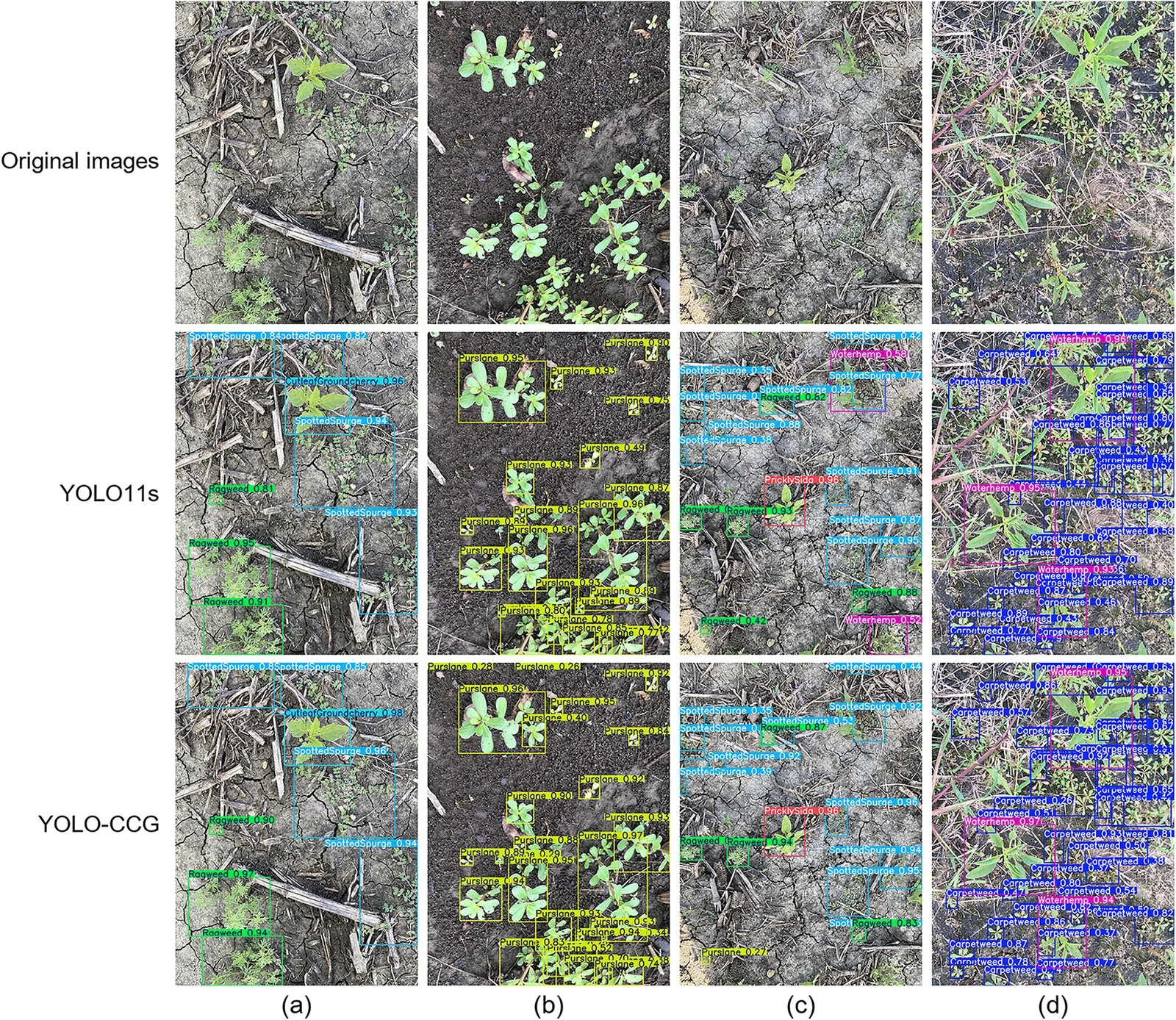
Visual comparison of detection results. (Top: original images; middle: YOLO11s; bottom: YOLO-CCG). (a) Complex background scenario; (b) Dense weed scenario; (c) Multi-species scenario; (d) Small target scenario.

#### 4.8.3. Feature heatmap visualization.

To visualize the feature responses of the proposed YOLO-CCG model, Grad-CAM++ [48] was applied to the corresponding intermediate feature layer in the baseline YOLO11s and improved YOLO-CCG models. The same input images and visualization settings were used for both models. In the resulting heatmaps, warm colors (red and yellow) indicate relatively higher normalized activation, whereas cool colors (blue and green) indicate relatively lower activation.

As shown in Fig 10, the strongest warm responses of both models are mainly located on the weed regions. Compared with the baseline YOLO11s model, YOLO-CCG shows more focused activation around the weed regions. These heatmaps provide qualitative evidence that YOLO-CCG can capture discriminative features of weeds under complex field conditions, complementing the quantitative detection results.

**Fig 10:**
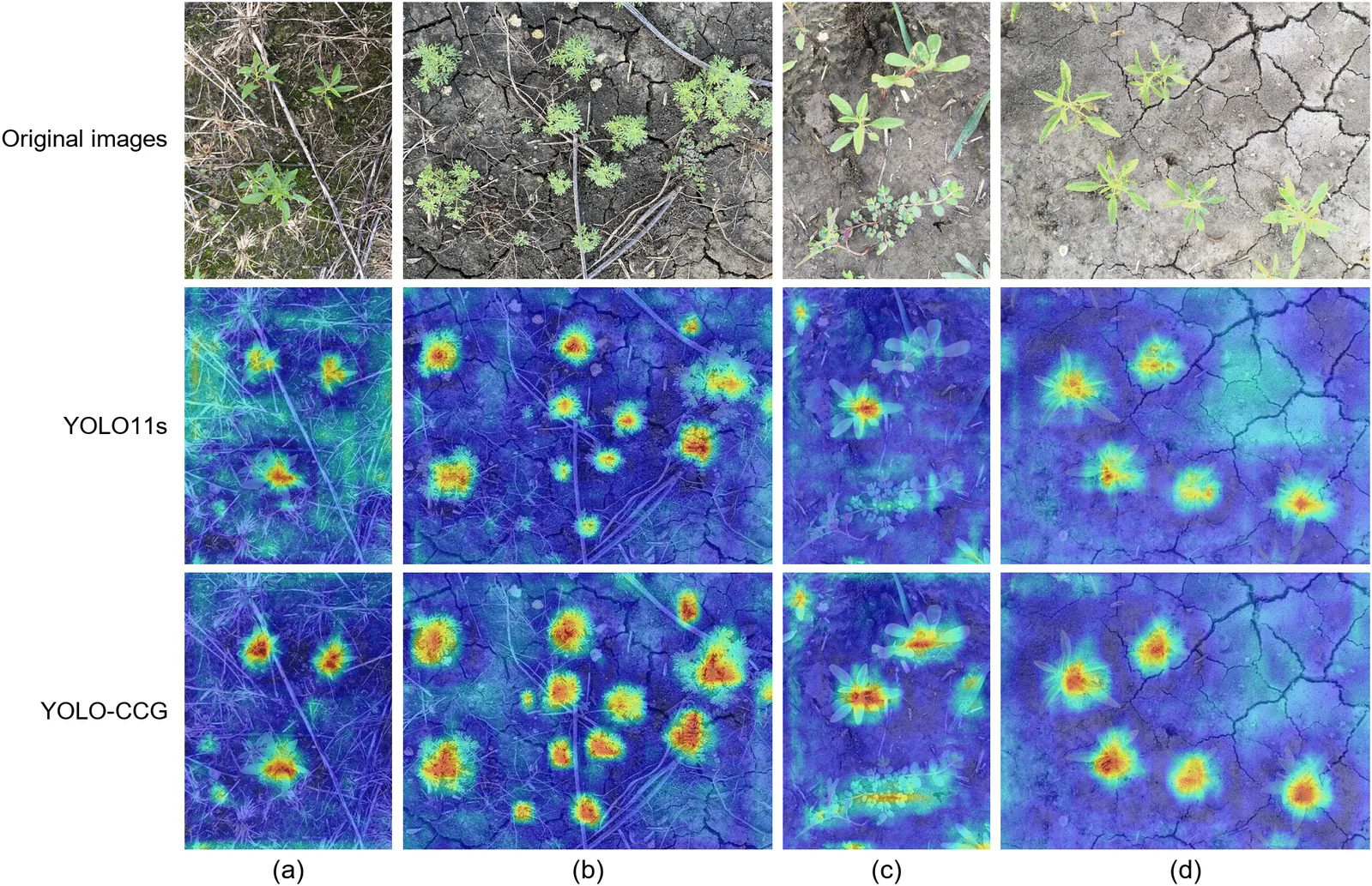
Grad-CAM++ heatmap visualization. (Top: original images; middle: YOLO11s; bottom: YOLO-CCG). (a) Complex background; (b) Dense weeds; (c) Multiple weed species; (d) Small weeds.

#### 4.8.4. Training curve analysis.

Fig 11 illustrates the training and validation loss curves of the baseline YOLO11s and the proposed YOLO-CCG throughout the training process. Both models exhibit stable convergence behavior, characterized by a rapid decline during the early training stage followed by gradual stabilization thereafter. No significant oscillation or divergence is observed, indicating that both models are effectively optimized. Compared with YOLO11s, YOLO-CCG generally exhibits lower training and validation losses. During the later training stages, the validation loss of YOLO-CCG remains stable without a noticeable rebound, indicating stable optimization behavior during the evaluated training process.

**Fig 11:**
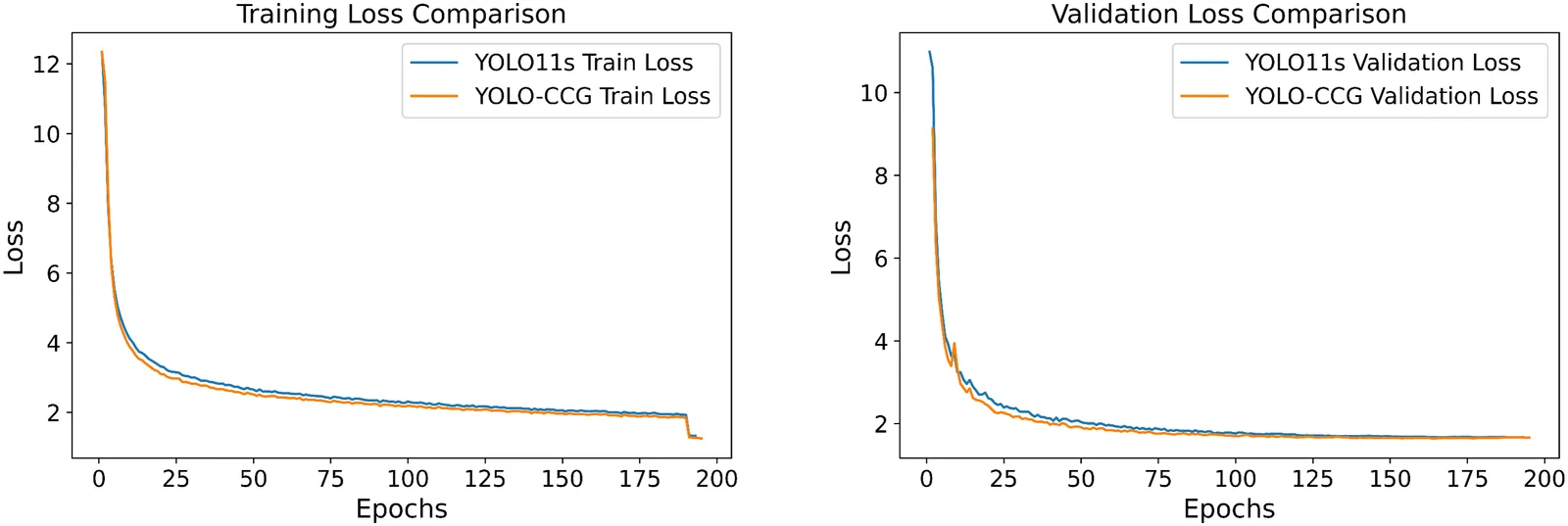
Training and validation loss curves of YOLO11s and YOLO-CCG.

### 4.9. Real-time detection system

A real-time cotton weed detection system was also developed with high flexibility and broad model compatibility. To support low-latency field deployment, the system provides four application modes: real-time camera capture, single-image precise analysis, continuous video stream processing, and folder-based batch detection.

To ensure operational efficiency, a decoupled multi-threaded architecture is adopted, where the main thread handles image acquisition and result visualization while a dedicated thread runs model inference. The system allows dynamic adjustment of the confidence threshold (0–1.0) and IoU threshold (0–1.0), as well as key frame capture. Detection sensitivity can be tuned according to cotton growth stages (e.g., seedling stage, budding stage) to minimize missed detections. The output can inform precise herbicide application, supporting the integration of detection, decision-making, and execution in intelligent weed management. The main interface is shown in Fig 12.

**Fig 12:**
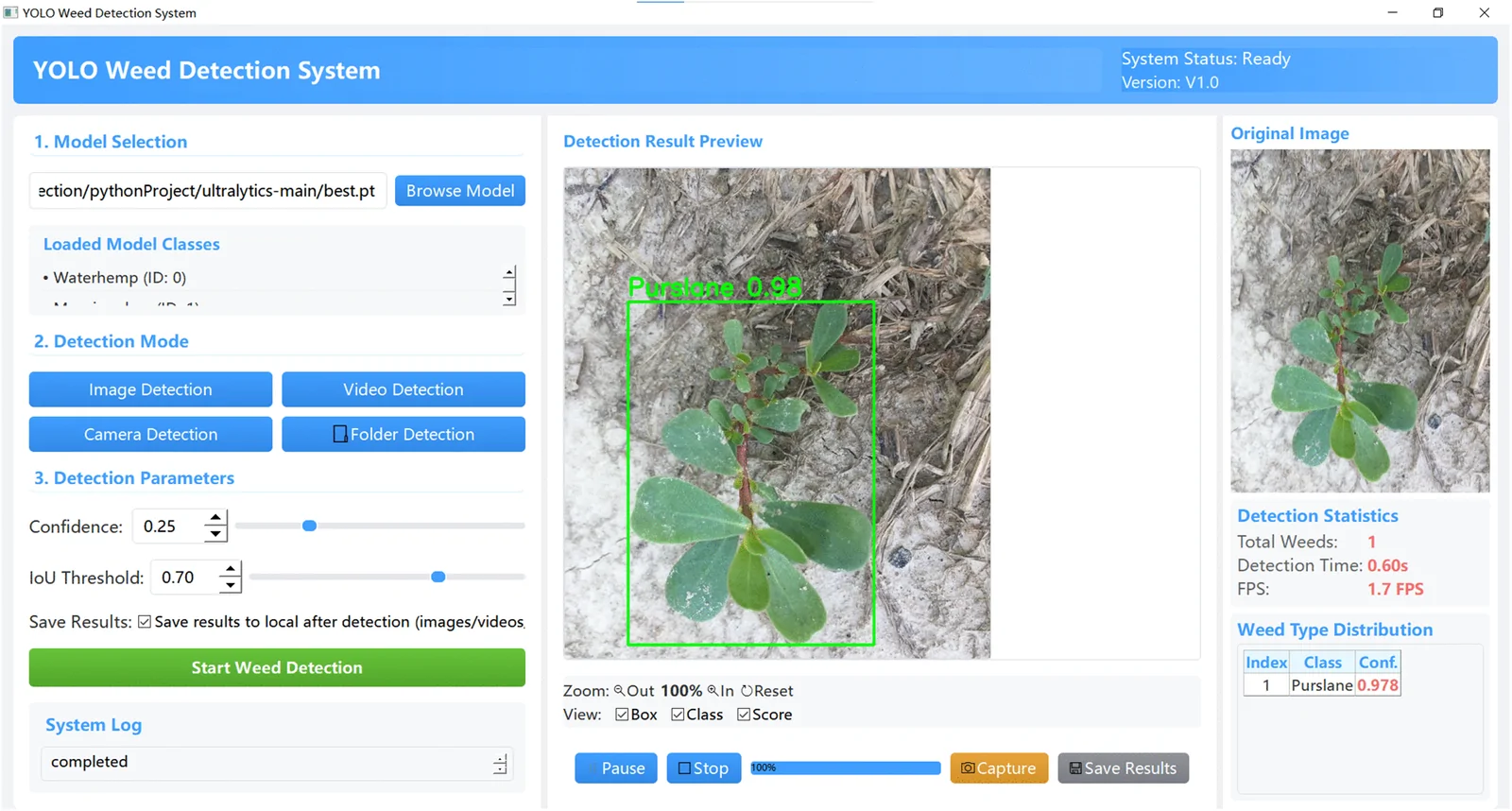
Main interface of the real-time weed detection system.

## 5. Conclusion

To mitigate false positives and missed detections in complex cotton-field environments, this study proposes an improved YOLO11-based detector named YOLO-CCG. The model integrates Coordinate Attention, CARAFE, GSConv, and VoVGSCSP to strengthen feature representation while controlling computational complexity.

Specifically, the CA module strengthens spatial and channel information modeling, enabling more accurate localization of weed targets. The CARAFE module uses content-aware reassembly during upsampling to retain fine-grained local information. Meanwhile, GSConv and VoVGSCSP reduce redundant computation in the corresponding bottom-up neck blocks while retaining feature fusion operations.

Experimental results on the CottonWeedDet12 dataset demonstrate that YOLO-CCG achieves a precision of 94.8%, recall of 90.0%, mAP50 of 95.1%, and mAP50-95 of 90.0%. Compared with the baseline YOLO11s model, precision, recall, mAP50, and mAP50-95 are improved by 0.4%, 2.2%, 0.8%, and 1.7%, respectively, in relative terms. Meanwhile, the computational complexity is reduced from 21.3 GFLOPs to 20.4 GFLOPs while maintaining a lightweight architecture with only 9.4 M parameters.

In addition, statistical analysis confirms that the improvements in precision, mAP50, and mAP50-95 are statistically significant (p < 0.05), demonstrating the effectiveness and reliability of the proposed architecture. Visualization results further reveal that YOLO-CCG can focus more accurately on weed regions and extract more discriminative features under complex backgrounds.

Although the proposed model achieves favorable performance, several limitations remain. First, the inference speed decreases slightly compared with the baseline model due to the introduction of additional feature enhancement modules. Second, the current experiments are conducted on a single cotton weed dataset, which may limit the model’s generalization ability across different crops and field environments.

Future work will focus on incorporating multimodal information such as infrared and hyperspectral imagery, developing adaptive attention mechanisms for dense weed scenarios, and applying model compression techniques including knowledge distillation and network pruning to facilitate deployment on resource-constrained agricultural edge devices. Furthermore, larger multi-crop weed datasets will be collected to improve model robustness and generalization performance in practical agricultural applications.

Code. https://github.com/gks-wlm/YOLOv11-CCG
